# Effectiveness comparison of nonpharmacological analgesia delivery methods: A protocol for systematic review and network meta-analysis

**DOI:** 10.1097/MD.0000000000022354

**Published:** 2020-09-18

**Authors:** Ying Li, Runmin Li, Yujin Yang, Yan Hu, Jia Xiao, Dongying Li

**Affiliations:** a Intensive Care Unit, the Second Affiliated Hospital of Nanchang University, Nanchang City, Jiangxi Province, Nanchang; b College of Traditional Chinese Medicine, Shandong University of Traditional Chinese Medicine, Jinan; c Nursing Department, the Second Affiliated Hospital of Nanchang University, Nanchang City, Jiangxi Province, Nanchang; d School of Nursing, Nanchang University, Jiangxi.

**Keywords:** delivery, network meta-analysis, nonpharmacological analgesia, protocol

## Abstract

**Background:**

Childbirth is a complex and special physiological process. Pain often accompanies the whole process of delivery. Long term pain will affect the physiological and psychological of pregnant women, and severe pain will affect the delivery process and the life of maternal and fetal. There are 2 ways to relieve delivery pain: drug analgesia and nonpharmacological analgesia. Nonpharmacological analgesia has less effect on the fetus than drug analgesia and is currently a more popular method for labor analgesia. Due to the lack of randomized trials comparing the efficacy of various nonpharmacological analgesia, it is still difficult to judge the relative efficacy. Therefore, we intend to conduct a network meta-analysis to evaluate the benefit among these nonpharmacological analgesia.

**Methods:**

According to the retrieval strategies, randomized controlled trials on nonpharmacological analgesia delivery will be obtained from China National Knowledge Infrastructure, WanFang,SinoMed, PubMed, Web of science, Embase, and Cochrane Library, regardless of publication date or language. Studies were screened based on inclusion and exclusion criteria, and the Cochrane risk bias assessment tool will be used to evaluate the quality of the literature. The network meta-analysis will be performed in Markov Chain Monte Carlo method and carried out with Stata14 and OpenBUGS14 software. Ultimately, the evidentiary grade for the results will be evaluated.

**Results:**

This study will provide more reasonable choice for clinic than the effect of nonpharmacological analgesia in parturient delivery.

**Conclusion:**

Our findings will provide references for future guidance developing and clinical decision.

INPLASY registration number: INPLASY202080097.

## 1 Introduction

Pregnancy is known as a critical and challenging period in women's lives because it is a process of transition that includes a variety of psychological, physiological, and social changes that occur simultaneously.^[1]^ The differences in women's perception of labor pain is the result of interactions between physiological and psychological aspects.^[2–4]^

Parturient women in the process of delivery: the first stage of labor features spasmodic contractions of the uterine smooth muscles and cervical dilatation due to physical stimulation, resulting in uterine and cervical ischemia, hypoxia, and a large number of hypoxic metabolites, which can stimulate the nerve endings of the reproductive tract and form nerve impulse nerves that transmit to the brain to form pain and radiate to the waist, abdomen, and buttocks. The pain in the second stage of labor is mainly due to the spasmodic contractions of the uterine smooth muscles and cervical dilatation, as well as the compression and expansion of the soft tissue of the rectum, pelvic floor, and perineum by the fetal head. The signals from the visceral and somatic nerve endings are transmitted to the central nervous system to form a somatic pain sensation. The pain in the third stage of labor is mainly the pain associated with placental separation.^[5,6]^

Delivery pain will lead to strong respiratory stimulation to the parturient woman, which will consume the physical strength of the parturient woman in the process of delivery and will lead to a significant increase in minute ventilation and oxygen consumption during uterine contractions.^[7]^ The excessive ventilation of pregnant women will lead to excessive carbon dioxide exhalation, resulting in severe respiratory alkalosis and a left shift of the maternal oxyhemoglobin dissociation curve, thus reducing the supply of oxygen to the fetus.^[8]^ Pain is experienced throughout the whole process of delivery, which seriously affects the progress of labor and the safety of both the mother and fetus.

Labor pain and methods to relieve it are a major concern for the mother and child, with considerable implications for intra- and postpartum care.^[9]^ At present, there are mainly drug analgesia and nonpharmacological analgesia for maternal labor. The commonly used drugs for labor analgesia include opioids, non-opioids, nitrous oxide, and patient-controlled analgesia (PCA). It has been reported that drug analgesia can cause adverse reactions such as nerve depression, respiratory depression, and heart rate slowing in newborns, and the long-term effects on newborns need to be systematically evaluated.^[10–13]^ In addition, for the mother, drug analgesia can cause a series of adverse reactions, such as pruritus, fever, drowsiness, nausea and vomiting, loss of consciousness, and respiratory disorders.^[7,13–16]^ The nonpharmacological therapies for pain relief include a variety of techniques, not only to relieve the physical sensations of pain but also to prevent suffering by enhancing the psychoemotional and spiritual components of care.^[17]^ The nonpharmacological analgesia are currently popular, mainly including music therapy, massage, doula, electric stimulation, training, hypnoses, acupuncture-moxibustion, and so on.

Although randomized controlled trials (RCTs) and meta-analysis have been frequently reported in studies of nonpharmacological analgesia delivery. However, due to the limitations of scale and research design, it is difficult to directly rank the efficacy of nonpharmacological analgesia. As a branch of traditional meta-analysis, network meta-analysis integrates the existing research and forms an evidence network that can indirectly compare the therapeutic benefits.^[18]^ This study will use network meta-analysis to evaluate the effectiveness and safety of different nonpharmacological analgesia for parturient delivery, and its conclusion will further guide clinical practice and strive for the best interests of patients.

## 2 Methods

### 2.1 Objectives and registration

This systematic review will aim to evaluate the effect and safety of CHI therapy for ACI. Our protocol has been registered on the International Platform of Registered Systematic Review and Meta-Analysis Protocols (INPLASY). The registration number was INPLASY202080097 (DOI:10.37766/inplasy2020.8.0097).

### 2.2 Ethics and communication plan

Our article is a secondary study, which does not involve the recruitment of patients, data collection, and ethical considerations. We will publish the results of network meta-analysis in the form of journal papers or conference papers.

### 2.3 Qualification criteria

According to the principle of PICOS, the inclusion and exclusion criteria of literature were determined.

#### 2.3.1 Types of participants

The diagnosis of parturient delivery is based on the Clinical Practice Guidelines Guidelines for Normal Delivery (2020)^[19]^ and Guidelines for Normal Delivery (2020).^[20]^ In order to reduce the occurrence of heterogeneity, all the subjects were primiparas.

#### 2.3.2 Types of interventions and controls

The control group was routine education and routine nursing. On the basis of conventional nursing care, the experimental group was added with analgesic measures. The commonly used nonpharmacological analgesia measures include: music therapy, massage, doula, electric stimulation, training, hypnoses, acupuncture-moxibustion, and so on.

#### 2.3.3 Types of outcomes

The primary outcomes should include the VAS score. Secondary outcomes will include the active stage of labor, the second stage of labor, postpartum hemorrhage volume, and neonatal Apgar score.

#### 2.3.4 Types of studies

The included studies will be RCTs in this systematic review regardless of publication status and language. Animal trials, systematic review, case reports and studies with incorrect designs or incomplete data will be excluded.

### 2.4 Data sources and retrieval strategy

Studies will be obtained from the China National Knowledge Infrastructure, Wan Fang Data, Chinese Scientific Journals Database (VIP), PubMed, CBM, Embase, Web of science and Cochrane Library, regardless of publication date or language. The databases will be retrieved by combining the subject words with random words. Taking PubMed as an example, the retrieval strategy is shown in Table 1. The search terms will be adapted appropriately to conform to the different syntax rules of the different databases.

**Table 1:**
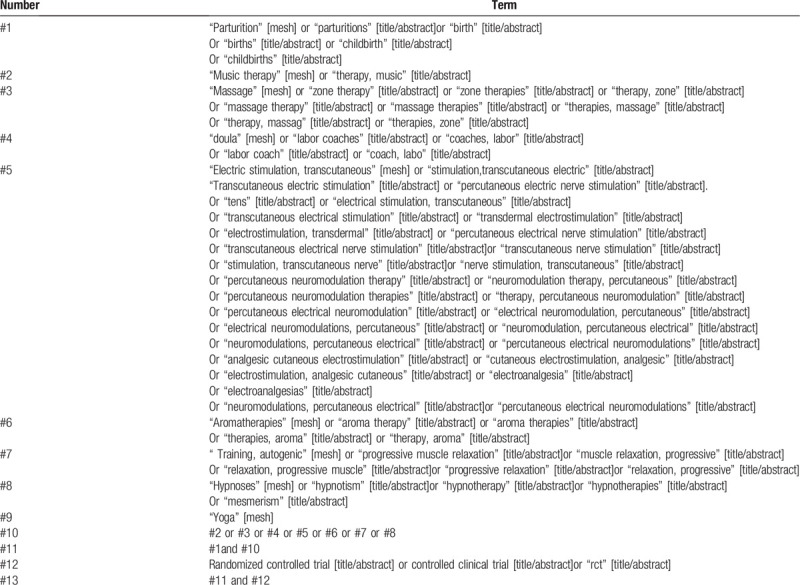
Retrieval strategy of PubMed.

### 2.5 Study selection and data extraction

**Figure 1:**
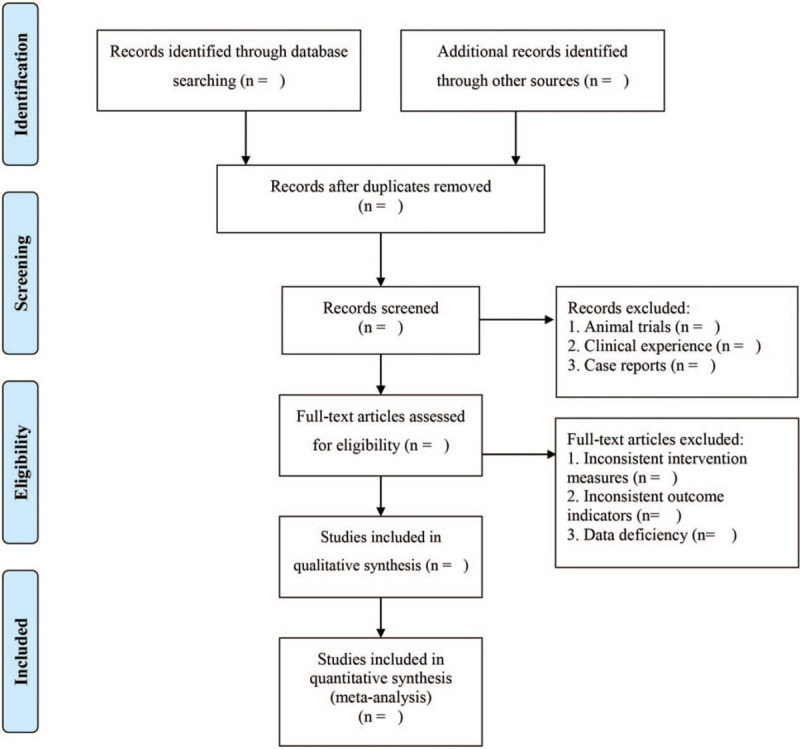
PRISMA flow chart.

EndNoteX9 will be used to manage the retrieved studies. As shown in Figure 1, the study selection will be divided into 2 steps and completed by 2 researchers (Jia Xiao and Yujin Yang). Preliminary screening: eliminate repeated and unqualified studies by reading the title and abstract. Rescreening: read through the full text and select the studies according to the inclusion and exclusion criteria. According to the Cochrane Handbook for Systematic Reviews of Interventions, the 2 researchers (Yan Hu and Jia Xiao) will extract the author, publication time, participant number, age, race, intervention measures in control group, Intervention measures in experimental group, course of treatment and outcome indicators, fill in the data extraction table, and compare the baseline levels of patients.

### 2.6 Risk of bias assessment

Two researchers (Yan Hu and Jia Xiao) assessed the quality of included RCTs independently by utilizing the Cochrane risk of bias assessment tool. As specified by Cochrane Handbook V.5.1.0, the following sources of bias were considered: random sequence generation, allocation concealment, participant blinding, intervention blinding, outcome assessor blinding, incomplete outcome data, selective reporting, and other sources of bias. Each domain was rated as having a high-risk, low-risk, or unclear-risk of bias as appropriate. The 2 reviewers resolved any differences through discussion. If no consensus can be reached, consult experts in the field and refer to their opinions.

### 2.7 Statistical analysis

#### 2.7.1 Traditional meta-analysis

Direct comparisons of nonpharmacological analgesia efficacy will be performed using Review Manager 5.3. The outcomes will be mainly represented by the mean difference or odds ratio with 95% confidence intervals. For continuous data, the pooled standarized mean differences (SMDs) and their corresponding 95% confindence intervals *95%* were used to assess the strength *P* < .05 was considered as statistically significant. The Cochrane Q-test and *I*^*2*^ statistics were used to assess heterogeneity. When *P* < .1 or *I*^*2*^ > 50%, which indicates statistical heterogeneity, a random-effects model will be used to calculate the outcomes; otherwise, a fixed-effects model will be considered.

#### 2.7.2 Network meta-analysis

A network evidence diagram will be drawn to visually represent the comparisons between the studies. The size of the nodes represents the number of participants, and the thickness of the edges represents the number of comparisons. Stata14 and OpenBUGS14 Software will be used to carry out Bayesian network meta-analysis. Bayesian inference will carried out using the Markov chain Monte Carlo method, the posterior probability will be inferred from the prior probability, and estimation and inference will be assumed when Markov Chain Monte Carlo reaches a stable convergence state. As a result, the grade of analgesic effect of different measures will be represented by the curve area or bar graph under the cumulative ranking curve.^[21]^

The node splitting method is used to evaluate the inconsistency between direct comparison and indirect comparison.^[22]^ The choices between consistent and inconsistent models and between fixed-and random-effect models will be made by comparing the deviance information criteria for each mode.^[23]^

#### 2.7.3 Subgroup and sensitivity analysis

If there is high heterogeneity in the included studies, we will perform subgroup analyses to explore the differences in age, sex, race, lesion location, and course of the intervention time. To ensure robustness of the combined results, sensitivity analyses will be performed to assess the impact of studies with a high risk of bias. We will compare the results to determine whether lower-quality studies should be excluded.

#### 2.7.4 Publication biases

We will use funnel plots to identify whether there will be small study bias if 10 or more studies are included. Asymmetry in the funnel plot will suggest the possibility of small study effects, and the results of analysis will be explained cautiously.

### 2.8 Quality of evidence

The grading of recommendations, assessment, development and evaluation approach will be used in evaluating evidence quality. Considerations of evidence quality assessment include study limitation, consistency of effect, imprecision, indirectness, and publication bias. The evidence quality will be classified into 4 levels (high, medium, low, and very low).^[24]^

## 3 Discussion

Although the current nonpharmacological analgesia is very concerned, but many nonpharmacological analgesia has not been fully clinical research and application. We call on more researchers to pay attention to the use of nonpharmacological analgesia. In addition, the choice of nonpharmacological analgesia should be based on strict experimental design and objective evaluation, and based on evidence. This study objectively evaluated a variety of non pharmacological analgesia, and provided guidance for clinical delivery.

## Author contributions

**Conceptualization:** Ying Li, Runmin Li, Dongying Li.

**Data curation:** Jia Xiao, Yujin Yang,Yan Hu.

**Formal analysis:** Ying Li, Runmin Li

**Methodology:** Dongying Li.

**Software:** Jia Xiao, Yujin Yang.

**Supervision:** Dongying Li.

**Writing – original draft:** Ying Li and Runmin Li.

**Writing – review & editing:** Dongying Li.

## Abbreviations

Abbreviation: RCTs = clinical randomized controlled trials.
